# ﻿Annotated checklist of arthropod-pathogenic species in the Entomophthoromycotina (Fungi, Zoopagomycota) in North America

**DOI:** 10.3897/mycokeys.114.139257

**Published:** 2025-03-05

**Authors:** Ann E. Hajek, Kelsey L. Scott, Sergio R. Sanchez-Peña, Cezary Tkaczuk, Brian Lovett, Kathryn E. Bushley

**Affiliations:** 1 Department of Entomology, Cornell University, Ithaca, NY 14853-2601, USA Cornell University Ithaca United States of America; 2 Emerging Pests and Pathogens Research Unit, USDA-ARS, Ithaca, NY 14853, USA Emerging Pests and Pathogens Research Unit, USDA-ARS Ithaca United States of America; 3 Departmento de Parasitología, Universidad Autónoma Agraria Antonio Narro, Saltillo, Coahuila, 25315, Mexico Universidad Autónoma Agraria Antonio Narro Saltillo Mexico; 4 Department of Horticulture and Plant Protection, University of Siedlce, 08-110 Siedlce, Poland University of Siedlce Siedlce Poland

**Keywords:** Biodiversity, entomopathogenic fungi, Entomophthorales, mycodiversity, pathogen species list

## Abstract

The subphylum Entomophthoromycotina (Phylum Zoopagomycota) includes many arthropod pathogens, some of which are renowned for their abilities to alter host behavior prior to death and cause epizootics that impact host populations. The last checklist of arthropod-pathogenic species in this group was published in 1963 and consisted of 39 species in a single genus. Since then, more species have been named, and their taxonomy has changed extensively. We have constructed an updated checklist for species of Entomophthoromycotina in North America; this checklist includes species in the continental United States, Canada, and Mexico. Data were compiled based on available published literature and metadata available from the ARSEF culture collection, adjusting names based on current taxonomy. In North America, the arthropod-pathogenic Entomophthoromycotina now include 80 species belonging to 14 genera, within two classes, plus one species in a form genus. This checklist provides a current framework for future studies of the biodiversity of this group of fungi.

## ﻿Introduction

Most species in the fungal subphylum Entomophthoromycotina (Phylum Zoopagomycota; Spatafora et al. 2016) are pathogens of arthropods. Many species in this group naturally cause epizootics (Pell et al. 2001), highlighting their potential use for biological control of arthropod pests. Species in this group are also renowned for altering the behavior of infected arthropod hosts in fixed, predictable patterns (de Bekker et al. 2021), including ‘summit behavior’ just prior to host death (e.g., Elya et al. 2018) or producing psychoactive metabolites to stimulate continued flight by spore-ejecting infected cicada hosts (Boyce et al. 2019).

Despite their promising roles in the control of insect pests and their fascinating biology, relatively few studies have documented the biodiversity, distribution, and ecology of entomophthoralean fungi in natural ecosystems. Most inventories of species of arthropod-pathogenic fungi in the Entomophthoromycotina originate from Europe. Twenty-six species have been listed for Spain (Niell and Santamaria 2001), 24 for Sweden (Gustafsson 1965), 12 for Norway (Klingen et al. 2002), 29 for Austria (Barta et al. 2005; Tkaczuk et al. 2011), and 35 for the United Kingdom (www.mapmate.co.uk/checklist). The much higher counts for Switzerland (95; Keller 2008; S. Keller pers. comm.) and Poland (94; Sosnowska et al. 2004; Mułenko et al. 2008; Dubiel 2016) are consistent with the suggestion that this group of fungi is most diverse in central Europe (Keller 2008). However, this statement might be biased by the prolific work of researchers specifically studying the subject in that geographical area. Outside of Europe, lists of species have also been reported for China (79; Zha et al. 2016), Israel (31; Ben-Ze’ev 1993), the Philippines (19; Villacarlos and Mejia 2004; Villacarlos 2008), Argentina (16; López Lastra et al. 2019), and Australia (10; Glare and Milner 1987), as well as in several different publications from Mexico (Remaudière and Latgé 1985; Sanchez-Peña 1990, 2000).

For North America, Roland Thaxter published a comprehensive monograph on the family Entomophthoraceae in the United States in 1888, in which he reported on 26 species (Thaxter 1888). Following this, Charles (1941) reported 36 species in this group for North America, and in 1963, Hutchison reported 39 species of entomophthoralean fungi that occurred in North, Central, and South America and provided information about hosts and geographic distributions of these species. In the 21^st^ century, a protochecklist of all nonlichenized fungi in North America included 38 species of arthropod-pathogens in the Entomophthoromycotina (Bates and Miller 2018). The protochecklist entries were based on herbarium specimens from the US (plus its territories), Canada, and Mexico, and in many instances, the names of arthropod pathogens that were included are not the currently accepted names.

Although there has not been an up-to-date summary of arthropod pathogens in North American species after 1963, the discovery, taxonomic changes, and research since that time have been prolific. It is very clear that an updated checklist is sorely needed; for example, the most recent protochecklist for all North American fungi does not use current taxonomic genera and species designations for this group (Bates and Miller 2018), certainly because this information might not be easy to find. Therefore, our goal with this annotated checklist was to present arthropod hosts and distributions for arthropod-pathogenic species in the Entomophthoromycotina that occur in North America, using the currently accepted taxonomy. This will facilitate continued progress in this field and summarize disparate observations as a compendium.

## ﻿Materials and methods

### ﻿Study area

This checklist of arthropod-pathogenic North American species includes records from the continental United States, Canada, and Mexico.

### ﻿The data

Published records that were used to begin generating this checklist were found in Thaxter (1888), Charles (1941), Hutchison (1963), and Bałazy (1993). The catalogue of the USDA Agricultural Research Service Collection of Entomopathogenic Fungi (ARSEF 2020) was also used to search for North American records falling within the arthropod-pathogenic Entomophthoromycotina. Subsequently, search engines were used to explore records for additional North American species, using a recent worldwide list of species within the arthropod pathogens of the Entomophthoromycotina (Sacco and Hajek 2023). Personal communication records for R.A. Humber are from his notes regarding specimens in the Thaxter collection at the Farlow Herbarium, Harvard University. Throughout the checklist, only naturally occurring fungi associated with field-collected arthropod hosts were included. Samples from laboratory manipulations, often from studies of host specificity, have not been included.

Within the Entomophthoromycotina, extensive taxonomic reorganization has occurred in relatively recent years, and many generic names have changed. For example, in 1963, *Entomophthora* is given as the generic name for all 39 species in the Western Hemisphere (Hutchison 1963). However, for all except 4 of these species, the generic names have since changed. We used Humber (1989), Index Fungorum (http://www.indexfungorum.org), and MycoBank (Robert et al. 2005) to assist with all taxonomic decisions about fungi. We only include information from records with fungal identifications to the species level. For all arthropod hosts, only the currently accepted genus and species names are used.

For all records, hosts and distributions are provided with associated citations. For distributional information for North America, we generally referred to states or provinces but also provided citations for references where more specific details about collection site locations were found. For some records, only the name of the country was available. In at least one publication, the locations of species by country were not provided, and this record therefore could not be included (e.g., Remaudière et al. 1978). Two-letter, capitalized abbreviations of US states and Canadian provinces were used. For records from Mexico, we used ‘conventional’ abbreviations that are 2–4 letters long for states (https://en.wikipedia.org/wiki/Template:Mexico_State-Abbreviation_Codes). In all cases, the country name occurs alongside states or provinces. GPS coordinates for sites are not included, as for most entries, references are older, and GPS information was not available. Alternatively, descriptions of sites were too vague or broad for us to infer a GPS location. GPS information provided in more recent reports is available via the citations provided.

For information about hosts, only pathogens in the Entomophthoromycotina infecting arthropods were included (Sacco and Hajek 2023). For example, reports of vertebrate infections by *Conidioboluscoronatus* and reports of species only isolated from soil (e.g., many *Conidiobolus* spp.) were not included.

Many arthropod-pathogenic species in the Entomophthoromycotina have not been isolated into pure culture. Among those that have, numerous have been frozen and deposited in culture collections. The USDA-ARS entomopathogenic fungi collection (ARSEF) is the world’s largest repository of these isolates, and we have indicated for each species whether a culture is available in ARSEF. We have not included GenBank accession numbers for numerous reasons, including that DNA data for most of these fungi are lacking or difficult to link to specimens, as many of these are older records prior to the DNA era. We can only confidently link accessions correctly to a few known specimens, and these are scarce. For those fungal species that have been sequenced, accession numbers can be found in the references cited or in GenBank.

References are always provided, but we did not exhaustively include references; if several references report a host/fungus association from the same state/province, we only included one of such references. Summaries were cited if these existed.

## ﻿Results

Records for host species and distributions are reported below. For species with more extensive lists of hosts and distributions, detailed accounts are included in tabular form. For example, for some species reported from many states across much of North America, hosts and distribution are summarized below, but individual host species and specific collection locations are listed in tables. Species names below followed by an asterisk have arthropod pathogenic North American isolates in the ARSEF culture collection.

### ﻿Class Neozygitomycetes

**Order Neozygitales**

**Family Neozygitaceae**

***Neozygites***

[**1] *Neozygitesfloridanus* (J. Weiser & Muma) Remaud. & S. Keller, 1980**

In the US, *N.floridanus* has been reported infecting six species of mites in the family Tetranychidae (Arachnida, Trombidiformes) in the southeastern US states, as well as IA, KS, PA, and TX (US) (Lopes Ribeiro et al. 2009) (Table 1). This species is frequently referred to as *N.floridana*, which is taxonomically incorrect.

**Table 1.** Recorded arthropod hosts of *Neozygitesfloridanus* in the US, all in the family Tetranychidae (Arachnida, Trombidiformes).

Host Species	Country	States	References
*Eotetranychussexmaculatus*	US	FL	Lopes Ribeiro et al. 2009
*Eutetranychusbanksi*	US	FL, TX	Pickett and Gilstrap 1986; Lopes Ribiero et al. 2009
*Oligonychuspratensis*	US	KS	Dick and Buschman 1995
*Panonychuscitri*	US	FL	Lopes Ribiero et al. 2009
*Tetranychusurticae*	US	AL, GA, IA, KS, MS, NC, NY, SC	Lopes Ribiero et al. 2009; ARSEF 2020
*Bryobia* sp.	US	PA	C. Tkaczuk, unpubl. data
-	US	PA	C. Tkaczuk, unpubl. data

[**2] *Neozygitesfresenii* (Nowak.) Remaud. & S. Keller, 1980**

**Figure 1.**
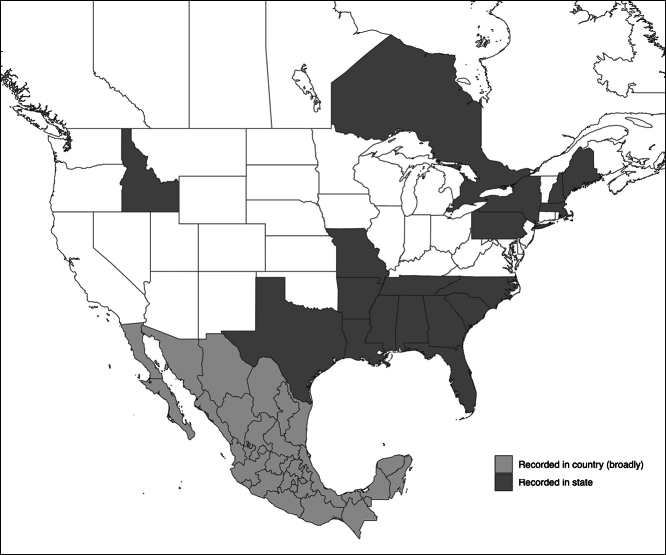
Distribution of recorded occurrences of *Neozygitesfresenii* in the US, Canada, and Mexico (see references in Table 2). Dark gray = locations by state in the US or province in Canada; light gray = a record from an unspecified area within Mexico.

On multiple aphid species (Hemiptera, Aphididae) from a broad distribution across the US, as well as ON (Canada) and Mexico (Fig. 1, Table 2).

**Table 2.** Recorded arthropod hosts of *Neozygitesfresenii* in the US, Canada, and Mexico, all hosts Hemiptera.

Host Family	Host Species	Country	States/Provinces	References
Aphididae	*Aphisglycines*	US	AR,NY	Nielsen and Hajek 2005; Galligan 2007
	*Aphisgossypii*	US	AL, AR, GA, LA, MS, MO, NC, SC, TN, TX	Sanchez-Peña 1993; Steinkraus et al. 1996
	*Aphisgossypii*	Mex	-	Remaudière and Latgé 1985
	*Aphispomi*	US	MA, ME, NC	Thaxter 1888
	*Aphissolitaria*	Mex	-	Remaudière and Latgé 1985
	*Aphisspiraecola*	US	FL	Charles 1941
	*Capitophoruselaeagni*	Mex	-	Remaudière and Latgé 1985
	*Myzuspersicae*	US	FL	Charles 1941
	*Periphylluslyropictus*	US	NH	Charles 1941
	*Schizaphisgraminum*	US	ID	Feng et al. 1990
	*Schizolachnuspiniradiatae*	Can	ON	Soper and MacLeod 1963
	-	US	PA	C. Tkaczuk, unpubl. data
Pseudococcidae	*Planococcuscitri*	US	LA	Charles 1941

[**3] *Neozygitesfumosus* (Speare) Remaud. & S. Keller, 1980**

On the mealybug *Planococcuscitri* (Hemiptera, Pseudococcidae) in FL and LA (US) (Speare 1922). This species is frequently referred to as *N.fumosa*, which is taxonomically incorrect.

[**4] *Neozygiteslageniformis* (Thaxt.) Remaud. & S. Keller, 1980**

Infecting ‘Aphides on *Betulapopulifolia*’ and ‘*Solidago*’ in MA, ME, and NC (US) (Thaxter 1888), and infecting *Macrosiphumeuphorbiae* and *Aphisnasturtii* (Hemiptera, Aphididae) in ME (US) (Shands et al. 1972).

[**5] *Neozygitesparvisporus* (D.M. MacLeod & K.P. Carl) Remaud. & S. Keller, 1980**

On *Frankliniella* sp. (Thysanoptera, Thripidae) in BC (Mexico) (Remaudière and Latgé 1985). This species is frequently referred to as *N.parvispora*, which is taxonomically incorrect.

[**6] *Neozygitesturbinatus* (R.G. Kenneth) Remaud. & S. Keller, 1980**

On *Cinaracurvipes* (Hemiptera, Aphididae, Lachninae) in Jal (Mexico) (Remaudière and Latgé 1985). This species is frequently referred to as *N.turbinata*, which is taxonomically incorrect.

### ﻿Class Entomophthoromycetes

**Order Entomophthorales**

**Family Conidiobolaceae**

***Conidiobolus***

[**7] *Conidioboluscoronatus* (Constantin) A. Batko 1964** *

On diverse insects, including termites (Isoptera, Kalotermitidae and Termitidae), aphids, and leafhoppers (Hemiptera), Diptera, Hymenoptera, and Thysanoptera. A truly polyphagous fungus, this species is also known to infect Collembola (Entognatha), Araneida, and Opiliones (Palpatores). It has been reported from across the US, Ver (Mexico), and AB (Canada) (Hutchison 1963; Matanmi et al. 1974; ARSEF 2020) (Table 3).

**Table 3.** Recorded arthropod hosts of *Conidioboluscoronatus* in the US, Canada, and Mexico.

Host Class	Host Order /Suborder	Host Family	Host Species	Country	States/ Provinces	References
Arachnida	Araneida	-	-	Can	AB	ARSEF 2020
	Opiliones/”Palpatores”	-	-	Can	AB	ARSEF 2020
Entognatha	Collembola	-	-	US	NC	ARSEF 2020
Insecta	Diptera	Anthomyiidae	*Deliaplatura*	US	WI	Matanmi et al. 1974
			*Deliaradicum*	US	WI	Matanmi et al. 1974
		Bibionidae	*Plecianearctica*	US	FL	Kish et al. 1974
		Sciaridae	*Lycoriellaingenua*	Mex	Ver	ARSEF 2020
	Hemiptera	Aphididae	*Aulacorthumsolani*	US	ME	Harris 1948
			*Macrosiphumeuphorbiae*	US	ME	Harris 1948
			*Metopolophiumdirhodum*	US	ID	ARSEF 2020
			*Myzuspersicae*	US	ME	Harris 1948
		Cercopidae	*Aeneolamiaalbofasciata*	Mex	Oax	Remaudiére and Latgé 1985
			*Aeneolamiacontigua*	Mex	Tamps	Guzmán and Alcocer-Gómez 1972
		Cicadellidae	*Empoascafabae*	US	NY	ARSEF 2020
	Hymenoptera	Formicidae	-	Can	AB	ARSEF 2020
	Isoptera	Kalotermitidae	-	US	CA, LA	Hutchison 1963
		Termitidae	-	US	CA, LA	Hutchison 1963
	Orthoptera	Acrididae	-	Mex	-	Remaudiére and Latgé 1985
	Thysanoptera	Thripidae	-	US	VT	ARSEF 2020
			*Frankliniellaoccidentalis*	US	FL	ARSEF 2020

### ﻿Family Neoconidiobolaceae

***Neoconidiobolus***

[8] *Neoconidiobolusthromboides* (Drechsler) B. Huang & Y. Nie, 2020*

Well known as an aphid (Hemiptera, Aphididae) pathogen: on numerous aphid hosts across the US and in AB, ON, and QC (Canada). It has also been reported from another hemipteran, the leafhopper *Empoascafabae* in NY (US) (Hemiptera, Cicadellidae), a heleomyzid fly in ME (US) (Diptera, Heliomyzidae), and an acridid in MT (US) (Orthoptera, Acrididae) (ARSEF 2020; Castrillo and Harris-Shultz 2024). *Deliaradicum* and *Deliaplatura* (Diptera, Anthomyiidae) were also reported infected in WI (US) (Matanmi et al. 1974) (Table 4).

**Table 4.** Recorded arthropod hosts of *Neoconidiobolusthromboides* in the US and Canada.

Host Order	Host Family	Host Species	Country	States/ Provinces	References
Diptera	Anthomyiidae	*Deliaplatura*	US	WI	Matanmi et al. 1974
		*Deliaradicum*	US	WI	Matanmi et al. 1974
	Heliomyzidae	-	US	ME	ARSEF 2020
Hemiptera	Aphididae	*Aphisglycines*	US	MN, NY	Nielsen and Hajek 2005
		*Diuraphistritici*	US	MT	Feng et al. 1991
		*Macrosiphumeuphorbiae*	US	ME	ARSEF 2020
		*Melanaphissacchari*	US	GA	Castrillo and Shultz 2024
		*Metopolophiumdirhodum*	US	ID	ARSEF 2020
		*Myzuspersicae*	US	FL, ME, WI	ARSEF 2020
		*Schizaphisgraminum*	Can	AB	ARSEF 2020
		*Sitobionavenae*	US	ID	ARSEF 2020
		*Therioaphismaculata*	US	CA	ARSEF 2020
		*Uroleucon* sp.	Can	QC	ARSEF 2020
	Cicadellidae	*Empoascafabae*	US	NY	ARSEF 2020
Orthoptera	Acrididae	-	US	MT	ARSEF 2020

### ﻿Family Batkoaceae

***Batkoa***

[9] *Batkoaapiculata* (Thaxt.) Humber, 1989*

Broad host range including four families of Lepidoptera (Erebidae, Noctuidae, Tortricidae, Geometridae), numerous aphid species (Hemiptera, Aphididae), leafhoppers (Hemiptera, Cicadellidae), a spittlebug (Hemiptera, Cercopidae), Scirtidae and Helotidae (Coleoptera), and nematocerans (Diptera). Collected across the northern US, as far south in the US as TN and NC but also in SLP (Mexico) (Thaxter 1888; Hutchison 1963; ARSEF 2020) (Table 5).

[10] *Batkoamajor* (Thaxt.) Humber, 1989*

Initially, described from Ptilodactylidae (Coleoptera) in NC (US) by Thaxter (1888). Epizootics occurred in *Lycormadelicatula*, an invasive species in the Fulgoridae (Hemiptera) in PA (US) in 2018 (Clifton et al. 2019). Following field surveys of host range in PA and NY (US), confirmed infections were observed in 3 families of Coleoptera, 8 families of Diptera, 5 families of Hemiptera, 6 families of Lepidoptera, and 1 family of Psocomorpha (Gryganskyi et al. 2022a) (Table 6).

**Table 5.** Recorded arthropod hosts of *Batkoaapiculata* in the US and Mexico.

Host Order	Host Family/Suborder	Host Species/Subfamily	Country	States	References
Coleoptera	Cantharidae	-	Mex	Oax	Remaudière and Latgé 1985
	Scirtidae	-	US	TN	Hutchison 1963
Diptera	Nematocera	-	US	ME, NC	Thaxter 1888
		-	Mex	Tamps	Remaudière and Latgé 1985
Hemiptera	Aphididae	*Acyrthosiphonpisum*	US	NY	ARSEF 2020
	Aphididae	*Macrosiphumeuphorbiae*	US	ME	ARSEF 2020
	Aphididae	*Myzuspersicae*	US	ME	ARSEF 2020
	Aphididae	*Rhopalosiphummaidis*	US	MT	ARSEF 2020
	Aphididae	*Rhopalosiphumpadi*	US	CO	ARSEF 2020
	Cercopidae	*Prosapiasimulans*	Mex	-	Remaudière and Latgé 1985
	Cicadellidae	-	US	NH, NY	ARSEF 2020
	Cicadellidae	*Typhlocyba* sp.	US	ME, NC	Thaxter 1888
Lepidoptera	Erebidae	*Hyphantriacunea*	US	ME, NC	Thaxter 1888
	Erebidae	listed as ‘Deltoid sp.’	US	ME, NC	Thaxter 1888
	Geometridae	*Petrophora* sp.	US	ME, NC	Thaxter 1888
	Tortricidae	*Tortrix* sp.	US	ME, NC	Thaxter 1888

**Table 6.** Recorded arthropod hosts of *Batkoamajor* in the US and Mexico.

Host Order	Host Family	Host Species	Country	States	References
Coleoptera	Cantharidae	*Rhagonychafraxini*	US	NY	Gryganskyi et al. 2022a
		*Rhagonycha* sp.	US	NY	Gryganskyi et al. 2022a
	Elateridae	*Athousbrightwelli*	US	NY	Gryganskyi et al. 2022a
	Ptilodactylidae	*Ptilodactylaserricollis*	US	NC	Thaxter 1888
	Tenebrionidae	*Isomirasericea*	US	NY	Gryganskyi et al. 2022a
Diptera	Anthomyiidae	-	US	NY	Gryganskyi et al. 2022a
	Dolichopodidae	*Gymnopterus* sp.	US	NY	Gryganskyi et al. 2022a
		*Medetera* sp.	US	PA	Gryganskyi et al. 2022a
		*Thrypticus* sp.	US	NY	Gryganskyi et al. 2022a
	Drosophilidae	*Drosophilasuzukii*	US	TN	ARSEF 2020
	Heleomyzidae	*Tephrochlamysrufiventris*	US	NY	Gryganskyi et al. 2022a
	Lauxaniidae	*Homoneurainserta*	US	NY, PA	Gryganskyi et al. 2022a
	Milichiidae	*Madizaglabra*	US	PA	Gryganskyi et al. 2022a
	Psychodidae	-	US	NY	Gryganskyi et al. 2022a
	Rhagionidae	-	US	NY	Gryganskyi et al. 2022a
		*Symphoromyia* sp.	US	NY	Gryganskyi et al. 2022a
	Sciaridae	-	US	NY, PA	Gryganskyi et al. 2022a
Hemiptera	Achilidae	-	US	NY	Gryganskyi et al. 2022a
	Aphididae	*Macrosiphumeuphorbiae*	US	ME	ARSEF 2020
	Cercopidae	*Prosapiasimulans*	Mex	Chis, Tamps	Remaudiére and Latgé 1985
	Cicadellidae	-	US	NY	Gryganskyi et al. 2022a
		*Empoascafabae*	US	NY	ARSEF 2020
	Cixiidae	*Cixius* sp.	US	NY	Gryganskyi et al. 2022a
	Derbidae	*Apachedegeeri*	US	NY	Gryganskyi et al. 2022a
	Fulgoridae	*Lycormadelicatula*	US	PA	Gryganskyi et al. 2022a
Lepidoptera	Blastobasidae	-	US	NY	Gryganskyi et al. 2022a
	Crambidae	*Eudonia* sp.	US	NY	Gryganskyi et al. 2022a
	Erebidae	*Lophocampacaryae*	US	NY	Gryganskyi et al. 2022a
		*Lymantriadispar*	US	NY	Gryganskyi et al. 2022a
	Geometridae	*Lambdinafiscellaria*	US	NY	Gryganskyi et al. 2022a
	Oecophoridae	*Fabiolaedithella*	US	NY	Gryganskyi et al. 2022a
	Tineidae	*Dryadaula* sp.	US	NY	Gryganskyi et al. 2022a
Psocoptera	Amphipsocidae	*Polypsocuscorruptus*	US	NY	Gryganskyi et al. 2022a
	Psocomorpha	-	US	PA	Gryganskyi et al. 2022a

[11] *Batkoaobscura* (I.M. Hall & P.H. Dunn) Gryganskyi, 2022*

On numerous species of aphids in CA, NY, ID, MT, and ME (US) and QC (Canada) (Table 7).

**Table 7.** Recorded arthropod hosts of *Batkoaobscura* in the US, Canada, and Mexico, all in the family Aphididae (Hemiptera).

Host Species	Country	States/ Provinces	References
*Acyrthosiphonpisum*	US	NY	ARSEF 2020
*Aphisnasturtii*	US	ME	Shands et al. 1972
*Aphis* sp.	Can	QC	ARSEF 2020
*Aulacorthumsolani*	US	ME	Shands et al. 1972
*Capitophoruselaeagni*	Mex	-	Remaudiére and Latgé 1985
*Capitophorusshepherdiae*	Mex	-	Remaudiére and Latgé 1985
*Capitophorusxanthii*	Mex	-	Remaudiére and Latgé 1985
*Hyperomyzuslactucae*	Mex	-	Remaudiére and Latgé 1985
*Macrosiphumeuphorbiae*	US	ME	Shands et al. 1962
*Macrosiphum* spp.	Mex	-	Remaudiére and Latgé 1985
*Metopolophiumdirhodum*	US	MT	Feng et al. 1991
Microparsus (Picturaphis) sp.	Mex	-	Remaudiére and Latgé 1985
*Myzuspersicae*	US	ME	Shands et al. 1962
*Myzuspersicae*	Mex	-	Remaudiére and Latgé 1985
*Rhopalosiphummaidis*	US	MT	Feng et al. 1991
*Rhopalosiphummaidis*	Mex	-	Remaudiére and Latgé 1985
*Rhopalosiphumpadi*	Can	QC	ARSEF 2020
*Rhopalosiphumpadi*	Mex	-	Remaudiére and Latgé 1985
*Rhopalosiphum* sp.	Mex	-	Remaudiére and Latgé 1985
*Therioaphismaculata*	US	CA	ARSEF 2020
*Uroleuconambrosiae*	Mex	-	Remaudiére and Latgé 1985
*Uroleuconsonchi*	Mex	-	Remaudiére and Latgé 1985
*Uroleucon* sp.	Can	QC	ARSEF 2020
*Uroleucon* sp.	Mex	-	Remaudiére and Latgé 1985

[**12] *Batkoapapillata* (Thaxt.) Humber, 1989**

On ‘several minute gnats’ (Diptera, Nematocera) from NH and NC (US). In North America, known only from these initial collections by Thaxter (1888).

### ﻿Family Entomophthoraceae

**Subfamily Entomophthoroideae**

***Arthrophaga***

[**13] *Arthrophagamyriapodina* K.T. Hodge & A.E. Hajek, 2017**

Reported from three species of millipedes (Myriapodina, Polydesmidae): *Apheloriavirginiensiscorrugata*, *Nannaria* sp., and *Borariainfesta* in MA, MD, NC, NY, PA, and VA, Washington DC (US), and southern ON (Canada) (Hodge et al. 2017).

#### ﻿*Entomophaga*

[14] *Entomophagaaulicae* (E. Reichardt) Humber, 1984 (*aulicae* species complex)*

*Entomophagaaulicae* is a complex of morphologically identical species infecting only Lepidoptera, within which only *E.maimaiga* has been named as a separate species (treated below). Aside from *E.maimaiga*, fungal populations belonging to the *E.aulicae* complex infect lepidopteran species in the Noctuidae, Erebidae, Geometridae, Tortricidae, Lasiocampidae, Notodontidae, Sphingidae, and Saturniidae families from around the US and Canada (FPMI 1990; Walsh 1996; Speare and Colley 1912; ARSEF 2020). An invasive species in the Hesperiidae (Lepidoptera) in QC (Canada) was also infected (McNeil and MacLeod 1982). Within the complex, genetic diversity has been detected, with two groups occurring in North America. Pathotype I includes hosts in the Geometridae, Tortricidae, Notodontidae, and Saturniidae from BC, ON, and NF (Canada) and ME, NY, and VT (US). Pathotype II includes species in the Noctuidae and Erebidae in GA, CA (US), and ON (Canada) (Hajek et al. 1996b; Walsh et al. 1990; Walsh 1996) (Table 8).

**Table 8.** Recorded arthropod hosts of *Entomophagaaulicae* in the US, Canada, and Mexico, with all hosts in the order Lepidoptera.

Host Family	Host species	Country	States/ Provinces	References	Group
Erebidae	*Catocala* sp.	US	CT	Speare and Colley 1912	-
	*Estigmeneacrea*	US	CT	Speare and Colley 1912	-
	*Estigmeneacrea*	Mex	Son	Young and Sifuentes 1959	-
	*Euchaetesegle*	US	-	Speare and Colley 1912	-
	*Euproctischrysorrhoea*	US	MA, ME	Kirkland 1906; Boyd et al. 2021	-
	*Hypenascabra*	US	SC	Kalkar and Carner 2005	-
	*Hyphantriacunea*	Can	ON	Walsh et al. 1990	II
	*Orgyiaantiquanova*	Can	NF	Thaxter 1888	-
	*Orgyialeucostigma*	Can	NS	van Frankenhuyzen et al. 2002	-
	*Orgyiavetusta*	US	CA	Hajek et al. 1996b	II
	*Pyrrharctiaisabella*	US	ME	Hitchings 1908	-
	*Spilosomavirginica*	US	OH, TX	Webster 1894; Mitchell 1919	-
Geometridae	*Epirritaautumnata*	Can	BC	FPMI 1990	-
	*Lambdinafiscellaria*	Can	BC, NF	Walsh et al. 1990; Hajek et al. 1996b	I
	*Nepytiafreemani*	Can	BC	FPMI 1990	-
	*Rheumapterahastata*	Can	ON	Walsh et al. 1990	I
	*Sabulodesgriseata*	Can	BC	ARSEF 2020	-
Lasiocampidae	*Malacosomaamericanum*	US	CT	Speare and Colley 1912	-
	*Malacosomadisstria*	US	NY	ARSEF 2020	-
Noctuidae	*Agrotis* sp.	US	CT	Thaxter 1891	-
	*Amphipyrapyramidoides*	US	-	Speare and Colley 1912	-
	*Helicoverpazea*	US	GA	Hamm 1980	-
	?*Heliothis* sp.	US	GA	Walsh et al. 1990	II
	*Heliothisvirescens*	US	GA	Hamm 1980	-
	*Lithophane* sp.	US	CT	Thaxter 1891	-
	*Mamestra* sp.	US	CT	Thaxter 1891	-
	*Mythimna* sp.	Can	ON	ARSEF 2020	-
	*Spodopterafrugiperda*	US	GA	Hamm 1980	-
Nolidae	*Nolacereella*	US	GA	Hamm 1980	-
Notodontidae	*Cecritabiundata*	Can	ON	ARSEF 2020	
	*Cecritaguttivitta*	US	NY, VT	Hajek et al. 1991; 1996b	I
	*Ellidacaniplaga*	Can	ON	ARSEF 2020	-
Saturniidae	*Dryocamparubicunda*	Can	ON	Walsh et al. 1990	I
Sphingidae	-	US	ME	FPMI 1990	-
	*Manducaquinquemaculata*	US	CT	Thaxter 1891	-
	*Manducasexta*	US	CT	Thaxter 1891	-
	*Pachysphinxmodesta*	US	ME	Farlow Herbarium unpubl. data	-
	*Eumorphafasciata*	US	FL	ARSEF 2020	-
Tortricidae	*Choristoneurafumiferana*	US	ME	Walsh et al. 1990	
	*Choristoneurafumiferana*	Can	NF, ON	Walsh et al. 1990	I
	*Choristoneuraoccidentalis*	Can	BC	Hajek et al. 1996b	I

[**15] *Entomophagabatkoi* (Bałazy) S. Keller, 1888**

On a species in the Phalangidae in ME (US) (R.A. Humber pers. comm.).

[16] *Entomophagacalopteni* (Bessey) Humber, 1989 (*E.grylli* species complex)*

A member of the *Entomophagagrylli* species complex, also known as Pathotype 2. Infects species in the Acrididae, with most infections in the subfamily Melanoplinae, although lower levels of infection have been found in the subfamilies Oedopodinae and Gomphocerinae. Reported from AZ, IA, KS, MT, ND, SD, and WY (US), AB and SK (Canada), and NL and Coah (Mexico) (Thaxter 1888; Soper et al. 1983; Erlandson et al. 1988; Bidochka et al. 1995, 1996; Casique-Valdes et al. 2012, 2022; ARSEF 2020).

[17] *Entomophagagrylli* Pathotype I (Fresen.) A. Batko, 1964 (*E.grylli* species complex)*

A member of the *Entomophagagrylli* species complex, also known as Pathotype 1 or *E.macleodii* (an unpublished name). Principally infects grasshopper species in the subfamily Oedopodinae (Acrididae), although also known to infect Gomphocerinae and occasionally Melanoplinae. Reported from AZ, ND, NY, MT, and OR (US), and AB, ON, and SK (Canada), and Coah (Mexico) (Bidochka et al. 1996; Casique-Valdes et al. 2012, 2022; Kistner and Belovsky 2013; ARSEF 2020).

[**18] *Entomophagakansana* (J.A. Hutchison) A. Batko, 1964**

Reported from Calliphoridae, Sarcophagidae, Muscidae, and Tachinidae (Diptera) near Lawrence, KS (US) (Hutchison 1962).

[19] *Entomophagamaimaiga* Humber, Shimazu & R.S. Soper, 1988 (*aulicae* species complex)*

**Figure 2.**
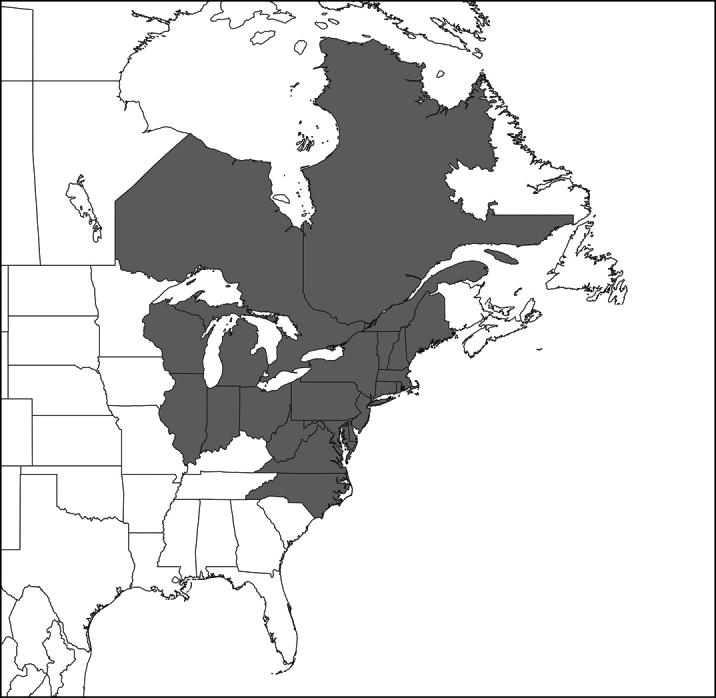
Distribution of recorded occurrences of *Entomophagamaimaiga* in the US and Canada, by state and province (Hajek et al. 2021; S. Picq pers. comm.).

*Entomophagamaimaiga* is a North Asian species only infecting Lepidoptera, accidentally introduced to North America, probably from Japan, at some time after 1971, but before 1989 (Weseloh 1998; Nielsen et al. 2005). Principal hosts are larvae of *Lymantriadispar* (Lepidoptera, Erebidae). Surveys of naturally occurring host specificity documented low levels of infections in 3 of 7 lymantriines (Hajek et al. 2004). Rare infections also occurred in *Catocalailia* (Erebidae) and *Malacosomadisstria* (Lasiocampidae) (Hajek et al. 1996a), *Agrocholabicolorago* (Noctuidae); an unidentified gelechiid (Lepidoptera) was also infected (Hajek et al. 2000). *E.maimaiga* was discovered in 1989 in seven northeastern US states (Hajek et al. 1990) and has since spread (naturally and with human assistance) into a total of 19 US states (Hajek et al. 2021). *E.maimaiga* has also been reported from ON and QC (Canada) (Nealis et al. 1999, S. Picq pers. comm.) (Fig. 2).

[**20] *Entomophagatabanivora* (J.F. Anderson & Magnar.) Humber, 1989**

On *Tabanusnigrovittatus* and *Atylotusthoracicus* (Diptera, Tabanidae) in MA and NY (US) (Anderson and Magnarelli 1979; Mullens et al. 1983).

[**21] *Entomophagatenthredinis* (Fresen.) A. Batko, 1964**

On sawfly larvae (Hymenoptera, Tenthredinidae), including *Pristiphoraerichsonii* in ON (Canada), larvae of an unidentified tenthredinid species in ME (US), and the introduced pine sawfly, *Diprionsimilis*, in WI (US) (Thaxter 1888; Klein and Coppel 1973; FPMI 1990).

#### ﻿*Entomophthora*

[**22] *Entomophthorachromaphidis* O.F. Burger & Swain, 1918**

An aphid pathogen (Hemiptera, Aphididae) on *Chromaphisjuglandicola* in CA (Burger and Swain 1918), the cereal aphids *Metopolophiumdirhodum* in ID, WA (US) and *Sitobionavenae* in ID (US) (Humber and Feng 1991), *Myzuspersicae* in ID (Kish et al. 1994), and *Aphisglycines* in NY (US) (Nielsen and Hajek 2005). Also reported on *Psocus* sp. (Psocodea, Psocidae) in CA (US) (Burger and Swain 1918). At least some of the entries under *E.planchoniana* (Table 9) could instead be *E.chromaphidis* (see Humber and Feng 1991; Barta and Cagáň 2006); further study is necessary.

[**23] *Entomophthoraculicis* (A. Braun) Fresen., 1858**

Reported by Thaxter (1888) on Diptera: “*Culex* [Culicidae], and numerous genera of minute flies or gnats” in MA, ME, and NH. On unidentified midges (Chironomidae) in NY (US) (Kramer 1981b) and unidentified black flies (Simuliidae) in AB (Canada) (Shemanchuk and Humber 1978).

[**24] *Entomophthoraerupta* (Dustan) I.M. Hall, 1959**

On Miridae (Hemiptera): *Neolyguscommunis*, *Adelphocorislineolatus*, *Irbisiasolani*, *Lygocorispabulinus*, and *Plagiognathus* sp. in NS (Canada) (Dustan 1924), and *I.solani* in CA (US) (Hall 1959). Also reported from *A.lineolatus* from NY (US) (Wheeler 1972).

[25] *Entomophthoramuscae* (Cohn) Fresen., 1856*

*Entomophthoramuscae* is a species complex of morphologically similar species infecting Diptera, including from 4 to 8 described species (Elya and De Fine Licht 2021). Three species within the complex occur in North America: *E.muscaesensu stricto* (s.s), *E.scatophagae* (treated separately), and *E.schizophorae* (treated separately). *Entomophthoramuscae* s.s infects *Muscadomestica* (Muscidae) in CA, NY, NC, and NE, and probably across most of the US and Canada, and in Coah (Mexico) (Sanchez-Peña unpubl. observations). Also on *Coenosiatigrina* (Muscidae) and *Deliaradicum* (Anthomyiidae) in NC (US) (Gryganskyi et al. 2013), and six species of *Drosophila* (Drosophilidae) in CA (US): *Drosophilamelanogaster*, *D.simulans*, *D.hydei*, *D.immigrans*, *D.pseudoobscura*, *D.repleta* (Elya et al. 2018). Further studies of hosts and distributions of the different members of this species complex in North America are necessary.

[**26] *Entomophthoraplanchoniana* Cornu, 1873**

On diverse species of aphids (Aphididae) across the US and Mexico (Table 9). However, at least some of the entries under *E.planchoniana* in Table 9 could instead be *E.chromaphidis* (see Humber and Feng 1991; Barta and Cagáň 2006); further study is necessary.

**Table 9.** Recorded arthropod hosts of *Entomophthoraplanchoniana* in the US and Mexico, with all hosts in the family Aphididae (Hemiptera).

Host Species	Country	States	References
*Acyrthosiphonmalvae*	Mex	-	Remaudière and Latgé 1985
*Acyrthosiphonpisum*	Mex	-	Remaudière and Latgé 1985
*Aphisasclepiadis*	Mex	-	Remaudière and Latgé 1985
*Aphiscoreopsidis*	Mex	-	Remaudière and Latgé 1985
*Aphisfabae*	Mex	-	Remaudière and Latgé 1985
*Aphisgossypii*	Mex	-	Remaudière and Latgé 1985
*Aphislugentis*	Mex	-	Remaudière and Latgé 1985
*Aphisnasturtii*	US	ME	Shands et al. 1972
*Aphissolitaria*	Mex	-	Remaudière and Latgé 1985
*Aphisspiraecola*	Mex	-	Remaudière and Latgé 1985
*Aulacorthumsolani*	US	ME	Shands et al. 1972
*Brevicorynebrassicae*	Mex	-	Remaudière and Latgé 1985
*Capitophoruselaeagni*	Mex	-	Remaudière and Latgé 1985
*Capitophorusshepherdiae*	Mex	-	Remaudière and Latgé 1985
*Capitophorusxanthii*	Mex	-	Remaudière and Latgé 1985
*Chaetosiphonfragaefolii*	US	CA	Dara 2017
*Cryptomyzusgaleopsidis*	US	ME	Shands et al. 1962
*Hayhurstiaatriplicis*	Mex	-	Remaudière and Latgé 1985
*Hyperomyzus* sp.	Mex	-	Remaudière and Latgé 1985
*Latgerinaorizabaensis*	Mex	-	Remaudière and Latgé 1985
*Macrosiphumeuphorbiae*	US	ME	Shands et al. 1962
*Macrosiphum* spp.	Mex	-	Remaudière and Latgé 1985
*Melanocalliscaryaefoliae*	US	GA	Ekbom and Pickering 1990
*Metopolophiumdirhodium*	US	ID	Feng et al. 1991
*Metopolophiumdirhodium*	Mex	BC	Remaudière and Latgé 1985
*Monelliacaryella*	US	GA	Ekbom and Pickering 1990
*Monelliopsispecanis*	US	GA	Ekbom and Pickering 1990
*Myzusornatus*	Mex	-	Remaudière and Latgé 1985
*Myzuspersicae*	US	ME	Shands et al. 1962
*Myzuspersicae*	Mex	-	Remaudière and Latgé 1985
*Obtusicauda* sp.	Mex	BC	Remaudière and Latgé 1985
*Rhopalosiphummaidis*	US	ID	Feng et al. 1991
*Rhopalosiphummaidis*	Mex	Coah	Sanchez-Peña 2000
*Rhopalosiphumpadi*	Mex	BC	Remaudière and Latgé 1985
*Rhopalosiphumpadi*	Mex	-	Remaudière and Latgé 1985
*Rhopalosiphum* sp.	Mex	-	Remaudière and Latgé 1985
*Sitobionavenae*	US	ME	Shands et al. 1962
*Uroleuconambrosiae*	Mex	-	Remaudière and Latgé 1985
*Uroleuconsonchi*	Mex	-	Remaudière and Latgé 1985
*Uroleucon* sp.	Mex	-	Remaudière and Latgé 1985
*Utamphorophoracrataegi*	Mex	-	Remaudière and Latgé 1985

[**27] *Entomophthorascatophagae* Giard, 1888**

A member of the *E.muscae* species complex (Jensen et al. 2006). On *Scatophagastercoraria* (Diptera, Anthomyiidae) in NY (Steinkraus and Kramer 1988).

[**28] *Entomophthoraschizophorae* S. Keller & Wilding, 1988**

A member of the *E.muscae* species complex, infecting only Diptera (Elya and De Fine Licht 2021). Isolates from NE and NY (US) infect *Polleniarudis* (Diptera, Polleniidae), and both also infect *Muscadomestica*, although at lower prevalence (Steinkraus et al. 1993a; Watson et al. 1993; Six and Mullens 1996). Also, infects *Hylemya* sp. (Anthomyiidae) in PA (US) (C. Tkaczuk unpubl. data).

#### ﻿*Eryniopsis*

[**29] *Eryniopsiscaroliniana* (Thaxter) Humber, 1984**

On *Tipula* sp. (Diptera, Tipulidae) in NC (US) (Thaxter 1888).

[**30] *Eryniopsislampyridarum* (Thaxter) Humber, 1984**

**Figure 3.**
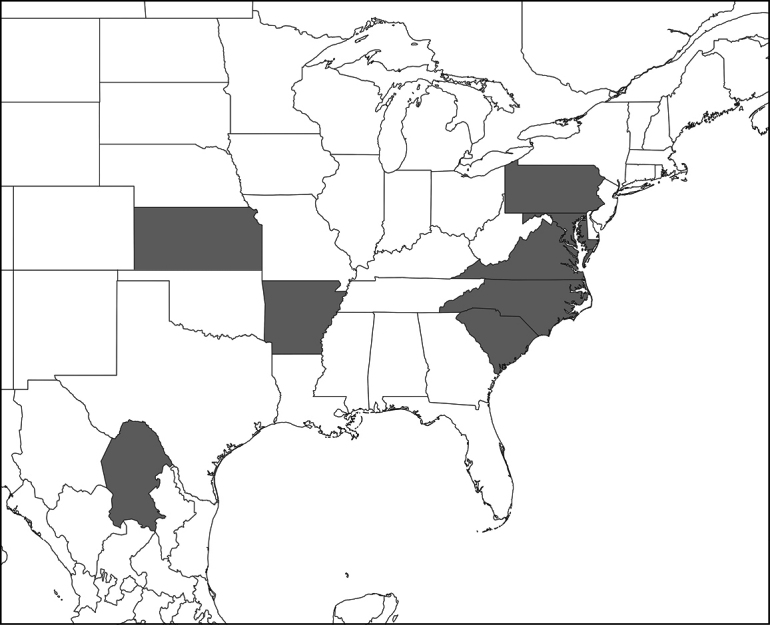
Distribution of recorded occurrences of *Eryniopsislampyridarum* in the US and Mexico, by state (see references in the text).

On *Chauliognathuspensylvanicus* and *Chauliognathusmarginatus* (Coleoptera, Cantharidae) in AR, MD, KS, NC, PA, SC, and VA (US) (Thaxter 1888; Carner 1980; Steinkraus et al. 2017) (Fig. 3). Also, on *Chauliognathus* sp. in Coah (Mexico) (RI Torres-Acosta & S. Sanchez-Peña, unpubl. observation: https://www.youtube.com/watch?v=NRBO7zc1J-o).

#### ﻿*Massospora*

[31] *Massosporacicadina* Peck, 1878*

On species of the genus *Magicicada* (Hemiptera, Cicadidae) in the eastern US (Macias et al. 2020).

[**32] *Massosporadiceroproctae* R.S. Soper, 1974**

On *Diceroproctadelicata*, *Diceroproctacinctifera*, *Diceroproctavitripennis*, and *Diceroproctabiconica* (Hemiptera, Cicadidae) in TX (and possibly LA, FL) (US) (Macias et al. 2020).

[**33] *Massosporafidicinae* R.S. Soper, 1974**

On *Fidicina* sp. (Hemiptera, Cicadidae) in Chis (Mexico) (Macias et al. 2020).

[**34] *Massosporalevispora* R.S. Soper, 1963**

On *Okanaganarimosa* and *Okanaganasperata* (Hemiptera, Cicadidae) in CA (US) and ON (Canada) and *Platypediaputnami* (Hemiptera, Cicadidae) in CA, NM, and UT (US) (Macias et al. 2020).

[**35] *Massosporaspinosa* Cif., A.A. Machado & Vittal, 1956**

On *Quesadagigas* (Hemiptera, Cicadidae) in NL (Mexico) (Macias et al. 2020).

#### ﻿*Orthomyces*

[**36] *Orthomycesaleyrodis* Steinkr., Humber & Oliv., 1998**

On *Trialeurodesabutiloneus* (Hemiptera, Aleyrodidae) in AL (US) (Steinkraus et al. 1998).

### ﻿Subfamily Erynioideae

***Erynia***

[37] *Eryniaaquatica* (J.F. Anderson & Ringo ex J.F. Anderson & Anagnost.) Humber, 1981*

On larvae and pupae of Culicidae (Diptera) in the US: *Aedescanadensis* (CT, RI), *Culisetamorsitans* (CT), *Aedesstimulans* (NY), *Aedesfitchii* (NY), *Aedescantator* (CT), *Aedes* sp. (NY) (Anderson and Ringo 1969; Molloy and Wraight 1982; Andreadis and Magnarelli 1983; Steinkraus and Kramer 1989; Christie 1996; ARSEF 2020).

[38] *Eryniaconica* (Nowak.) Remaud. & Hennebert, 1980*

On Chironomidae, Chaoboridae, Simuliidae, and Tipulidae (Diptera) in NC, NH, and NY (US) (Thaxter 1888; Cuebas-Incle 1992; ARSEF 2020). On Simuliidae (Diptera) in QC (Canada): *Simuliumvenustum* complex, *Simuliumverecundum/rostratum*, *Simuliumvittatum* complex (Nadeau et al. 1994).

[39] *Eryniacurvispora* (Nowak.) Remaud. & Hennebert, 1980*

Reported from adult *Simuliumdecorum* (Diptera, Simuliidae) in NY (US) and QC (Canada) (Kramer 1983; Nadeau et al. 1994), *Aedestriseriatus* (Diptera, Culicidae) in ME (US), and unidentified *Simulium* in NY (US) and QC (Canada) and Trichoptera in QC (Canada) (ARSEF 2020).

[**40] *Eryniagracilis* (Thaxter) Remaud. & Hennebert, 1980**

On “very minute gnats” (Diptera) in NC (US) (Thaxter 1888).

[**41] *Eryniaovispora* (Nowak.) Remaud. & Hennebert, 1980**

On a ‘small gnat attached to bark’ in TN (Diptera) (US) (R.A. Humber pers. comm.).

[42] *Eryniarhizospora* (Thaxter) Remaud. & Hennebert, 1980*

On Neuroptera and “several genera” of adult Phryganeidae (Trichoptera) in ME and NC (US) (Thaxter 1888). Also reported on Trichoptera in NY (US) (ARSEF 2020).

[43] *Eryniasepulchralis* (Thaxter) Remaud. & Hennebert, 1980*

On unidentified adult crane flies (Diptera, Tipulidae) in western NC and eastern TN (US) (Thaxter 1888), and on *Tipulacaloptera* in NY (US) (Kramer 1980).

[**44] *Eryniavariabilis* (Thaxter) Remaud. & Hennebert, 1980**

On ‘minute gnats of various genera’ in NC (Diptera) (US) (Thaxter 1888).

#### ﻿*Pandora*

[**45] *Pandoraamericana* (Thaxter) S. Keller, 2007**

On Diptera: *Muscadomestica* (Muscidae), *Calliphoravomitoria*, and *Luciliacaesar* (Calliphoridae) and ‘numerous other large flies.’ Common in New England and less common in NC (US) (Thaxter 1888). Also reported in the US from *Calliphora* sp. (WI), *C.vomitoria* (AL), *Phormiaregina* (TX) (Calliphoridae), and *Muscinastabulans* (TN) (Muscidae) (Charles 1941).

[**46] *Pandorablissi* (G. Lakon) D.M. MacLeod & Müller-Kögler, 1973; *nomen dubium***

On the chinch bug, *Blissusleucopterus* (Hemiptera, Blissidae) in IA, IL, KS, MN, and OH (Billings and Glenn 1911). This species was initially placed in the genus *Empusa* by Thaxter (Gillette 1888). The 1888 species description was minimal (Gillette 1888), and no types were designated (but ‘co-types’ are present at Harvard). The provisional genus *Pandora* is based on ongoing evaluation of museum specimens.

[47] *Pandorablunckii* (G. Lakon ex G. Zimm.) Humber, 1989*

On *Plutellaxylostella* (Lepidoptera, Plutellidae) in Gto. (Mexico) (ARSEF 2020) and on Diptera (possibly Sciaridae) in NY (US) (ARSEF 2020).

[**48] *Pandorabullata* (Thaxt. & D.M. MacLeod ex Humber) Humber, 1989**

On Calliphoridae (Diptera): including *Phormiaregina*, *Luciliasericata*, *Protophormiaterraenovae*, *Calliphoravomitoria*, and perhaps other *Calliphora* spp. and Sarcophagidae (Diptera): *Sarcophagaaldrichi* in ON (Canada) and NY, MI, and MA (US) (MacLeod et al. 1973; Kramer 1979; Nielsen et al. 2001).

[49] *Pandoradelphacis* (Hori) Humber, 1989*

On Hemiptera: *Spissistilusfestinus* (Hemiptera, Membracidae) in AL, AR (US) (Miller and Harper 1987; ARSEF 2020), and *Empoascafabae* (Hemiptera, Cicadellidae) and Miridae in NY (ARSEF 2020).

[50] *Pandoradipterigena* (Thaxter) Humber, 1989*

On Diptera: “small Tipulidae; other small flies or gnats belonging especially to the Mycetophilidae” in MA, ME, NC, and NH (US) (Thaxter 1888), unknown dipterans in ME and NY (US) (ARSEF 2020), and nematocerans in Mich. (Mexico) (Remaudière and Latgé 1985).

[**51] *Pandoraechinospora* (Thaxter) Humber, 1989**

On *Minettiaduplicata* (Diptera, Lauxaniidae) and ‘rarely other smaller Diptera’ in ME, NH, and NC (US) (Thaxter 1888).

[**52] *Pandoraformicae* (Humber & Bałazy) Humber, 1989**

On Formicinae (Hymenoptera, Formicidae) in ME (US) (R.A. Humber pers. comm.).

[**53] *Pandoragammae* J. Weiser ex. Humber, 1989**

On noctuid larvae (Lepidoptera): *Chrysodeixisincludens* in AL and GA (US) in GA (US) and Tamps (Mexico) and *Trichoplusiani* in AL (US) and Coah (Mexico) (Harper and Carner 1973; Newman and Carner 1975; Gilreath et al. 1986; Sanchez-Peña 1990, 2000). Also on *Mocis* sp. in NL and Coah (Mexico) (S.R. Sanchez-Peña unpubl. data).

[54] *Pandoragastropachae* (Racib.) Hajek & Gryganskyi, 2024*

In hardwood forests on *Malacosomadisstria* (Lepidoptera, Lasiocampidae), ranging from BC to QC (Canada) and ME to FL and AL (US) (Filotas et al. 2003).

[**55] *Pandoragloeospora* (Vuillemin) Humber, 1989** *

On *Lycoriellamali* (Diptera, Sciaridae) in mushroom production facilities in MD, DE, and southeastern PA (US) (Miller and Keil 1990, AP Gryganskyi pers. comm.) and cadavers producing conidia found on oyster mushrooms (*Pleurotus* sp.) in FL (US) (MW Miller pers. comm.).

[56] *Pandoraheteropterae* (Bałazy) S. Keller, 2005*

On *Lyguslineolaris* (Hemiptera, Miridae) in AR (US) (Hannam and Steinkraus 2010).

[57] *Pandoraithacensis* (Kramer) Hajek & Gryganskyi, 2024*

On Diptera: *Symphoromyiahirta* and *Rhagiomystaceus* (Rhagionidae) and *Empisobesa* (Empididae) in NY (US) (Kramer 1981a) and an unidentified rhagionid in PA (US) (C. Tkaczuk unpubl. data).

[**58] *Pandoramontana* (Thaxter) Hajek & Gryganskyi, 2024**

On Diptera on the alpine summit of Mt. Washington, NH (US), infecting “minute gnats, apparently *Chironomus* sp.” (Thaxter 1888).

[**59] *Pandoramuscivora* (J. Schröt.) S. Keller, 2005**

On *Syrphus* sp. (Diptera, Syrphidae) in ME (US) (MyCoPortal 2024).

[60] *Pandoraneoaphidis* (Remaud. & Hennebert) Humber, 1989*

On diverse aphids (Hemiptera, Aphididae) across the US, Mexico, and QC (Canada). In addition, single records of *Lygus* sp. (Hemiptera, Miridae) in NY (US) (ARSEF 2020) and *Aeneolamiaalbofasciata* (Hemiptera, Cercopidae) in Mexico (Remaudière and Latgé 1985) (Table 10). These last records of this fungus on spittlebugs and mirids should be reconsidered due to the unambiguous nature of this fungus as a specialized aphid pathogen, and the brief and rather incomplete description in these records.

**Table 10.** Recorded arthropod hosts of *Pandoraneoaphidis* in the US, Canada, and Mexico, all hosts in the order Hemiptera.

Host Family	Host Species	Country	States/ Provinces	References
Aphididae	*Acyrthosiphonkondoi*	US	CA	Pickering and Gutierrez 1991
	*Acyrthosiphonpisum*	US	CA, GA, ID, IL, MN, NY, PA, WA	Folsom 1909; Pickering et al. 1989; Pickering and Gutierrez 1991; ARSEF 2020; C. Tkaczuk unpubl. data
	*Acyrthosiphonpisum*	Mex	-	Remaudière and Latgé 1985
	*Aphisasclepiadis*	Mex	-	Remaudière and Latgé 1985
	*Aphiscoreopsidis*	Mex	-	Remaudière and Latgé 1985
	*Aphisfabae*	US	WA	ARSEF 2020
	*Aphisfabae*	Mex	-	Remaudière and Latgé 1985
	*Aphisglycines*	US	NY	ARSEF 2020; Nielsen and Hajek 2005
	*Aphisgossypii*	Mex	-	Remaudière and Latgé 1985
	*Aphislugentis*	Mex	-	Remaudière and Latgé 1985
	*Aphissolitaria*	Mex	-	Remaudière and Latgé 1985
	*Aphis* sp.	Can	QC	ARSEF 2020
	*Aphis* sp.	US	WA	ARSEF 2020
	*Aphisspiraecola*	Mex	-	Remaudière and Latgé 1985
	*Aulacorthumsolani*	US	ME	Shands et al. 1972
	*Brachycaudushelichrysi*	Mex	-	Remaudière and Latgé 1985
	*Brachyunguistetrapteralis*	Mex	-	Remaudière and Latgé 1985
	*Brevicorynebrassicae*	Mex	-	Remaudière and Latgé 1985
	*Capitophoruselaeagni*	Mex	-	Remaudière and Latgé 1985
	*Capitophorusshepherdiae*	Mex	-	Remaudière and Latgé 1985
	*Capitophorusxanthii*	Mex	-	Remaudière and Latgé 1985
	*Cavariellahendersoni*	Mex	-	Remaudière and Latgé 1985
	*Diuraphisnoxia*	US	CO, ID	ARSEF 2020; Feng et al. 1990
	*Hayhurstiaatriplicis*	Mex	-	Remaudière and Latgé 1985
	*Hyperomyzuslactucae*	Mex	-	Remaudière and Latgé 1985
	*Hyperomyzus* sp.	Mex	-	Remaudière and Latgé 1985
	*Illinoia* sp.	Mex	-	Remaudière and Latgé 1985
	*Impatientinumamericanum*	Mex	-	Remaudière and Latgé 1985
	*Macrosiphumeuphorbiae*	US	FL, ID, ME	ARSEF 2020; Feng et al. 1990
	*Macrosiphum* spp.	Mex	-	Remaudière and Latgé 1985
	*Melanaphissacchari*	Mex	Coah	ARSEF 2020
	*Metopolophiumdirhodum*	US	ID, MT	ARSEF 2020; Feng et al. 1990, 1991
	*Myzusornatus*	Mex	-	Remaudière and Latgé 1985
	*Myzuspersicae*	US	AR, ID, ME, VA, WA	Harris 1948; Elkassabany et al. 1992; Kish et al. 1994; Dara and Semtner 2001
	*Myzuspersicae*	Mex	-	Remaudière and Latgé 1985
Aphididae	*Myzuspersicaenicotianae*	US	KY, VA	ARSEF 2020; Dara and Semtner 2001
	*Rhapalosiphumpadi*	US	ID	Feng et al. 1990
	*Rhodobiumporosum*	Mex	-	Remaudière and Latgé 1985
	*Rhopalosiphummaidis*	US	ID, MT	Feng et al. 1990, 1991
	*Rhopalosiphummaidis*	Mex	-	Remaudière and Latgé 1985
	*Rhopalosiphumpadi*	Mex	-	Remaudière and Latgé 1985
	*Rhopalosiphum* sp.	Mex	-	Remaudière and Latgé 1985
	*Schizaphisgraminum*	US	ID	ARSEF 2020; Feng et al. 1990
	*Schizaphisgraminum*	Mex	-	Remaudière and Latgé 1985
	*Sibobionavenae*	US	ID	Feng et al. 1990
	*Sitobion* sp.	Mex	-	Remaudière and Latgé 1985
	*Uroleuconambrosiae*	Mex	-	Remaudière and Latgé 1985
	*Uroleuconsonchi*	Mex	-	Remaudière and Latgé 1985
	*Uroleucon* sp.	Can	QC	FPMI 1990
	*Uroleucon* sp.	Mex	-	Remaudière and Latgé 1985
	*Utamphorophoracrataegi*	Mex	-	Remaudière and Latgé 1985
	*Wahlgreniellaarbuti*	Mex	-	Remaudière and Latgé 1985
Cercopidae	*Aenolamiaalbofasciata*	Mex	-	Remaudière and Latgé 1985
Miridae	*Lygus* sp.	US	NY	ARSEF 2020

[**61] *Pandoranouryi* (Remaud. & Hennebert) Humber, 1989** *

On aphids (Hemiptera: Aphididae) on potato in ME (US) (ARSEF 2020).

[62] *Pandorapieris* (Z.Z. Li & Humber) Hajek & Gryganskyi, 2024*

On larvae of *Pierisrapae* (Lepidoptera, Pieridae) in NY (US) (Li and Humber 1984).

[**63] *Pandorasylvestris* Hajek & Gryganskyi, 2024**

On larvae of *Lophocampacaryae* (Lepidoptera, Erebidae) from MI and VT (US) (Hajek et al. 2024a).

[**64] *Pandoravirescens* (Thaxter) Hajek & Gryganskyi, 2024**

On Noctuidae (Lepidoptera): *Mythimnaunipuncta* in AR (US), *Dargidaprocinctus* in OR (US), and *Ochropleurafennica* in ON (Canada) (Steinkraus et al. 1993b).

[**65] *Pandoravomitoriae* (Rozsypal) Hajek & Gryganskyi, 2024**

On adult Calliphoridae (Diptera): ‘blue bottle flies’ in Coah. (Mexico) (Sanchez-Peña 2000) and *Luciliasericata* in NY (US) (B. Lovett unpubl. data).

***Zoophthora***

[**66] *Zoophthoraaphrophorae* (Rostr.) S. Keller, 2007**

On the pine spittlebug, *Aphrophoraparallela* (Hemiptera, Aphrophoridae), in PA (US) (Knull 1932).

[**67] *Zoophthoracanadensis* (MacLeod, Tyrrell & Soper) Remaud. & Hennebert, 1980**

On *Schizolachnuspiniradiatae* (Hemiptera, Aphididae) in red pine plantations in ON (Canada) (MacLeod et al. 1979).

[**68] *Zoophthoraforficulae* (Giard) A. Batko, 1964**

On *Forficulaauricularia* (Dermaptera, Forficulidae) in OR and WA (US) (Rockwood 1950; Hutchison 1963).

[**69] *Zoophthorageometralis* (Thaxt.) A. Batko, 1964**

On adults of *Eupithecia* sp., *Petrophora* sp., and *Thera* sp. (Lepidoptera, Geometridae) in ME (US) (Thaxter 1888).

[**70] *Zoophthoraichneumonis* Bałazy, 1993**

On an adult ichneumonid (Hymenoptera) in PA (US) (C. Tkaczuk, unpubl. data).

[**71] *Zoophthoraindependentia* A.E. Hajek, Humber & Gryganskyi, 2016**

Resting spore stages occurred within adult *Tipulasubmaculata* (Diptera, Tipulidae) in NY (US) (Hajek et al. 2016).

[72] *Zoophthoraoccidentalis* (Thaxter) A. Batko, 1964*

First reported on ‘aphides on *Betulapopulifera*’ in MA and ME (US) (Thaxter 1888). On aphids (Hemiptera, Aphididae), *Myzuspersicae*, *Macrosiphumeuphorbiae*, and *Aphisfabae* in ME (US); *Acyrthosiphonpisum* and *Aphisglycines* in NY (US); and *Sitobionavenae* in ID (US) (Feng et al. 1990; Nielsen and Hajek 2005; Barta and Cagáň 2006; ARSEF 2020).

[**73] *Zoophthoraphalloides* A. Batko, 1966**

Aphid pathogens (Hemiptera, Aphididae) on *Macrosiphumeuphorbiae*, *Nearctaphisbakeri*, *Uroleucon* sp., and *Acyrthosiphonpisum* in QC (Canada), ME and NH (US) (Remaudiére et al. 1978). On *A.pisum* in Oax. (Mexico) and *Therioaphismaculata* in Mexico (ARSEF 2020) and NY (US) (Milner and Soper 1981).

[74] *Zoophthoraphytonomi* (Arthur) A. Batko, 1964*

**Figure 4.**
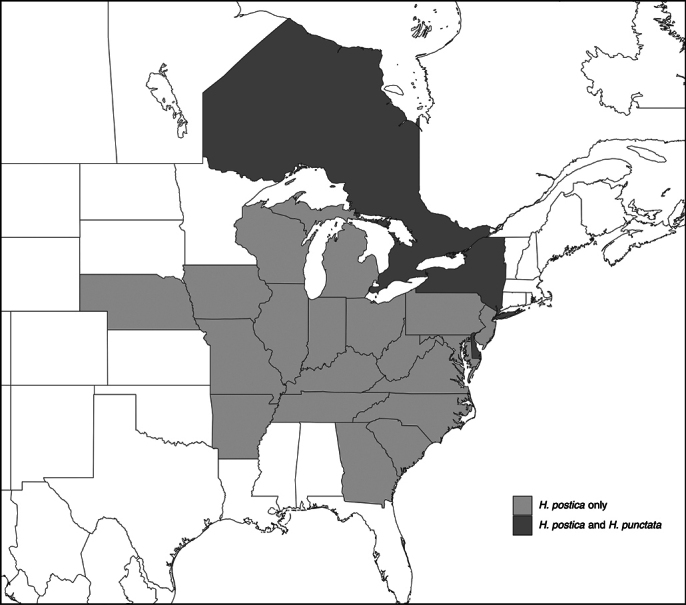
Distribution of recorded occurrences of *Zoophthoraphytonomi* in the US and Canada (Hajek et al. 1995). Dark gray = states and provinces where this pathogen was reported from both *Hyperapostica* and *Hyperaphytonomi*; light gray = US states where this pathogen was only reported from *H.postica* (Hajek et al. 1995).

Two genotypes infecting weevils in the genus *Hypera* (Coleoptera, Curculionidae) occur in North America (Hajek et al. 1995). The genotype principally infecting *Hyperapostica* was first found in 1973 in ON (Canada) and subsequently in 21 eastern US states (Fig. 4). This genotype was also reported in *H.punctata* from NY (US). The second genotype infected *Hyperapunctata* in ON (Canada) and NY and DE (US) (Hajek et al. 1995).

[**75] *Zoophthoraporteri* (R.S. Soper) A.E. Hajek, Humber & Gryganskyi, 2016**

Resting spore stages occurred within adult *Tipulacolei* (Diptera, Tipulidae) in TN (US) (Hajek et al. 2016).

[76] *Zoophthoraradicans* (Bref.) A. Batko, 1964*

On hosts in diverse families across Hemiptera, Lepidoptera, Hymenoptera, and Diptera. Widespread distribution across the US, Canada, and Mexico (Table 11).

**Table 11.** Recorded arthropod hosts of *Zoophthoraradicans* in the US, Canada, and Mexico.

Host Order	Host Family	Host Species	Country	States/ Provinces	References
Diptera	Drosophilidae	-	US	FL	ARSEF 2020
	Nematocera	-	Mex	Dgo	Remaudière and Latgé 1985
	Tipulidae	-	US	ME	FPMI 1990
Hemiptera	Aphididae	*Acyrthosiphonpisum*	US	GA	Pickering et al. 1989
		*Aphisnasturtii*	US	ME	Shands et al. 1972
		*Macrosiphumeuphorbiae*	US	ME	Shands et al. 1962
		*Metopolophiumdirhodum*	US	ID, MT	Feng et al. 1990
		*Myzusornatus*	Mex	-	Remaudière and Latgé 1985
		*Sitobionavenae*	US	ID	Feng et al. 1990
		*Therioaphismaculata*	US	NY	ARSEF 2020
		*Therioaphismaculata*	Mex	CDMX	FPMI 1990
		*Therioaphistrifolii*	Mex	CDMX	Remaudière and Latgé 1985
	Cicadellidae	*Empoascafabae*	US	IL, MI, NY, WI	ARSEF 2020; McGuire et al. 1987
	Pentatomidae	*Bagradahilaris*	Mex	Coah	Torres-Acosta et al. 2016
	Psyllidae	*Trioza* sp.	Can	QC	ARSEF 2020
		*Psyllatrimaculata*	Can	QC	FPMI 1990
		*Cacopsyllamali*	Can	NS	Gilliatt 1925
	Triozidae	*Bactericeracockerelli*	Mex	Coah	Torres-Acosta et al. 2016
Hymenoptera	-	-	US	ME	ARSEF 2020
	Diprionidae	*Neodipriontsugae*	US	AK	ARSEF 2020
Lepidoptera	Geometridae	*Lambdinafiscellaria*	Can	NF	Otvos et al. 1973
	Hesperiidae	*Thymelicuslineola*	Can	QC	FPMI 1990
	Noctuidae	*Autographaprecationis*	US	IN	Yendol and Paschke 1987
		*Rachiplusiaou*	US	IN	Yendol and Paschke 1987
		*Trichoplusiani*	US	IN	Yendol and Paschke 1987
		*Trichoplusiani*	Mex	Coah	Sanchez-Peña 2000
	Plutellidae	*Plutellaxylostella*	Mex	Gto	ARSEF 2020
	Tortricidae	*Aclerisvariana*	US	ME	ARSEF 2020
		*Aclerisvariana*	Can	NF	FPMI 1990
		*Archipsargyrospila*	US	PA	Knull 1932
		*Choristoneurabiennis*	Can	BC	FPMI 1990
		*Choristoneurafumiferana*	US	ME	Vandenberg and Soper 1975
		*Choristoneurafumiferana*	Can	BC, NS, ON	FPMI 1990
		*Rhopobotanaevana*	US	MA	Sawyer 1933

[**77] *Zoophthorarhagonycharum* (Bałazy) S. Keller, 2007**

Resting spore stages in adult *Rhagonychavilis* and *Rhagonychafraxini* (Coleoptera, Cantharidae) in NY (US) (Hajek et al. 2024b).

#### ﻿*Strongwellsea*

[**78] *Strongwellseacastrans* A. Batko & Weiser, 1965**

On *Deliaplatura* (Diptera, Anthomyiidae) in WI (US) (Strong et al. 1960) and *Deliaradicum* in ON (Canada) (Nair and McEwen 1973).

[**79] *Strongwellseamagna* Humber, 1976**

On *Fanniacanicularis* (Diptera, Fanniidae) in CA (US) (Humber 1976).

### ﻿FORM GENUS

***Tarichium***

[**80] *Tarichiummegaspermum* Cohn, 1875**

On two species of Noctuidae (Lepidoptera): *Euxoamessoria* and *Euxoaochrogaster* in BC and ON (Canada) (Bucher and MacLeod 1974; Steinkraus et al. 1993b).

### ﻿Incomplete and questionable records

For some species we could not resolve the identification of the fungal species, especially older identifications based only on morphology or species for which confusion exists regarding the correct fungal species name to use. For example, the species *Entomophthoracarpentieri*, named by Giard in 1888 from only resting spores collected in Europe, was identified by V.K. Charles from *Horistonusuhleri* (Coleoptera, Elateridae) collected in 1934 by J.N. Tenhet in SC (MyCoPortal 2024). However, this fungal species is considered questionable by Keller (1991) and Bałazy (1993), and any potential synonymous species are not known in North America, and so it has not been included in this checklist.

One isolate of *Entomophagaconglomerata* is listed in the ARSEF 2020), but a recent publication demonstrated that this isolate instead belongs in the genus *Batkoa* (Gryganskyi et al. 2022b), and therefore this example was not included. Tipulids in NHand NC (US), collected by Thaxter, harbored a fungus named *Entomophthorathaxteri* (MacLeod and Müller-Kögler 1973), but this species was not adequately described. However, trying to find the correct genus and species for this species has been difficult. Over time, it has been suggested that this species could be *E.conglomerata*, *Entomophagatipulae*, or *Entomophthoratipulae* (MacLeod and Müller-Kögler 1973; Bałazy 1993). In addition, because Thaxter found only resting spores in adults, perhaps this is *Zoophthoraindependentia* or *Z.porteri*. Therefore, this species (whatever it is) is not included in the checklist. In the case of *Pandorablissi*, this genus name is provided as *nomen dubium*; the native host of this species is no longer as abundant as in the past (Waldbauer 2005), and studies are currently underway to obtain specimens so the correct genus can be determined.

Sometimes, pathogen/host associations seem incorrect in initial reports. For example, in 1909, *Zoophthoraradicans* was reported infecting the weevil *Hyperapunctata* in IL (US) (Folsom 1909). However, now we know that *Zoophthoraphytonomi* (not described until 1964) infects this weevil species in other regions (Fig. 4), and we have no examples of *Z.radicans* infecting Coleoptera (see Table 11). In this case, we have not included this location information under *Z.phytonomi* (Fig. 4) or under *Z.radicans* (Table 11).

Finally, as we do not include entries for which a species name has not been provided. Thus, we could not include an unidentified species infecting the economically important northern corn rootworm, *Diabroticabarberi* (Naranjo and Steinkraus 1988); in this case beetle cadavers only contained resting spores. We also could not include the only example from the Arctic. *Zoophthora* sp. was reported causing epizootics in outbreak populations of the noctuid *Euroisocculta* in West Greenland (Avery and Post 2013). The larval cadavers that had summited on the vegetation only contained resting spores, and the species could not be identified or described at that time.

## ﻿Discussion

In 1963, a checklist of entomophthoralean fungi from the Western Hemisphere listed 39 species, all ascribed to the same genus: *Entomophthora* (Hutchison 1963). Today, with increased collection data and the incorporation of taxonomic changes within this group, our survey of arthropod pathogens in the Entomophthoromycotina found in North America includes 80 species in 14 genera, within 2 classes, plus one species in a form genus. A recent checklist of all fungi in North America (Bates and Miller 2018) lists only 38 arthropod-pathogenic species belonging to the Entomophthoromycotina. This checklist most likely relied on the Hutchison (1963) checklist, as it was the most recent available checklist for this group (Hutchison, 1963), underscoring the necessity for an updated checklist of Entomophthoromycotina in North America.

In their checklist for all North American fungi, Bates and Miller (2018) discuss the issue that there are many instances where European names were initially assigned to North American taxa based on morphological similarities. However, North American strains could represent cryptic taxa native to this continent that should be described as separate species, particularly if supported by molecular findings. We assume that this type of work will be undertaken in the future. However, at present many species in this group have not been isolated *in vitro* and/or there are no herbarium samples that can be used for DNA extraction. Therefore, to move forward along these molecular lines, in many cases, new specimens must be collected for analyses.

In fact, many species of arthropod pathogenic Entomophthoromycotina found worldwide have not been isolated in culture, and sequences are not available for molecular identification. In nature, viable cells of these fungi are quite ephemeral, and it is therefore difficult to collect them for isolation. The exception to this would be cadavers bearing resting spores (azygospores or zygospores). However, resting spores are often not found or, if found, are dormant and difficult to either germinate or use for DNA extraction (but see Bidochka et al. 1995; Eilenberg and Jensen 2018; Hajek et al. 2018). The ARSEF collection has North American cultures or samples from 29 of the species included in this checklist (approximately 36%). We hope that this annotated checklist will make it more possible in the future for the culture and sequencing of additional species in North America.

Taxonomic changes in this group have been relatively frequent since the first publication on species of Entomophthoromycotina in the United States by Thaxter in 1888, referring to this group as the Entomophthoreae. These taxonomic changes pose challenges for understanding whether names for host/fungus associations in the older literature are accurate today. For example, *E.muscae* is now known to be a species complex (Elya and De Fine Licht 2021), with one species named *E.muscae* s.s delimited within the complex. Therefore, for older records when *E.muscae* is mentioned, it is unclear which of the species in the complex is being discussed and how to apply historical findings to the modern circumscription of the species *E.muscae* itself. Therefore, since this checklist treats the three species of this species complex known from North America separately, the older reports of *E.muscae* have usually not been included in order not to introduce errors. The *Entomophagagrylli* species complex raised similar problems. Today, two members of this complex occur in North America, and older literature did not differentiate between them, with the result that older reports for collection locations could not be included.

Likewise, confusion has occurred with *Pandoraneoaphidis*, for which the correct nomenclature was only resolved in 1980 (Barta and Cagáň 2006). From 1888 (Thaxter) until 1980, in North America, *P.neoaphidis* was incorrectly known as *Empusaaphidis* and later *Entomophthoraaphidis*. However, in 1980 *Entomophthoraaphidis* was synonymized with *Zoophthoraaphidis*, a species known only from relatively few host aphids in western and central Europe. We assume that records of *Empusa* or *Entomophthoraaphidis* in North America before 1980 probably refer to *P.neoaphidis*, especially for collections from Maine, due to the common occurrence of *E.aphidis* and the presence of cultures of *P.neoaphidis* from Maine collected in 1972 and 1977 in the ARSEF 2020).

Our records of the distribution of fungal species are predominantly based on reports in the literature. The time of year, or even year of collection, is not always reported. Additionally, entomophthoralean fungi have been collected by only a handful of experts and remain vastly understudied across many locations in North America. Therefore, if a state or province is not listed, this is not definitive proof that a species is not present there, but rather this suggests that further surveys are necessary.

This annotated checklist provides data on these arthropod pathogens by connecting arthropod host species and fungal species at different locations within North America. Therefore, for each record, three specific pieces of information were needed: 1) host species, 2) fungal species, and 3) collection location. Unfortunately, some publications (although relatively few) did not provide separate data for these three metadata types and thus could not be included. Such problems arose with Remaudière et al. (1978), where all fungal pathogens and their aphid hosts were merged for one trip covering NH (US), and ON and QC (Canada). Similarly, we could not include data from Feng et al. (1990), in which data from collections of three species of aphid-pathogenic *Conidiobolus* species were merged so that locations and hosts of individual species were not listed (one of these species is now a *Batkoa*), or from Wraight et al. (1993), in which data for fungal infections of several cereal aphid species were merged.

## ﻿Conclusion

In conclusion, we provide an updated checklist of arthropod pathogenic fungi in the Entomophthoromycotina detected in North America, using the latest taxonomy and largely based on published literature. While this checklist includes many more species than the last checklist (Hutchison 1963), there are still many arthropod-pathogenic species known from this subphylum (see Sacco and Hajek 2023) that have not been found in North America. Additional sampling is needed to determine if these are truly absent from the continent or await discovery as well as whether new discoveries await. Additionally, given the paucity of molecular data (De Fine Licht et al. 2016), sequencing of additional species from verified specimens, together with improved taxonomies, is an area of future research that will enhance DNA-based identification of these fungi. This updated checklist provides a framework for future efforts sampling and documenting the biodiversity of this important, yet understudied, group of fungi.

## ﻿Additional information

### Author ORCIDs

Ann E. Hajek https://orcid.org/0000-0001-5740-4717

Kelsey L. Scott https://orcid.org/0000-0003-1378-5348

Sergio R. Sanchez-Peña https://orcid.org/0000-0002-1505-8657

Cezary Tkaczuk https://orcid.org/0000-0002-4096-2154

Brian Lovett https://orcid.org/0000-0002-5721-7695

Kathryn E. Bushley https://orcid.org/0000-0002-9083-8602

### Data availability

All of the data that support the findings of this study are available in the main text.
